# Antihypertensive effects of yoga in a general patient population: real-world evidence from electronic health records, a retrospective case-control study

**DOI:** 10.1186/s12889-022-12569-3

**Published:** 2022-01-27

**Authors:** Nadia M. Penrod, Jason H. Moore

**Affiliations:** 1 Department of Biostatistics, Epidemiology, and Informatics at the University of Pennsylvania, D202 Richards Building, 3700 Hamilton Walk, Philadelphia, PA 19104–6116 USA; 2 Institute for Biomedical Informatics, Perelman School of Medicine, University of Pennsylvania, D202 Richards Building, 3700 Hamilton Walk, Philadelphia, PA 19104–6116 USA; 3 Department of Computational Biomedicine, Cedars-Sinai Medical Center, Los Angeles, CA USA

**Keywords:** Yoga, Blood pressure, Electronic health records, Integrative medicine, Population health

## Abstract

**Background:**

Despite decades of research and established treatment strategies, hypertension remains a prevalent and intractable problem at the population level. Yoga, a lifestyle-based practice, has demonstrated antihypertensive effects in clinical trial settings, but little is known about its effectiveness in the real world. Here, we use electronic health records to investigate the antihypertensive effects of yoga as used by patients in their daily lives.

**Methods:**

A retrospective, observational case-control study of 1815 records among 1355 yoga exposed patients and 40,326 records among 8682 yoga non-exposed patients collected between 2006 and 2016 from a regional academic health system. Linear mixed-effects models were used to estimate the average treatment effect of yoga on systolic and diastolic blood pressures. Mixed effects logistic regression models were used to calculate odds ratios for yoga use and four blood pressure categories: normal, elevated, stage I, and stage II hypertension.

**Results:**

Yoga patients are predominantly white (88.0%) and female (87.8%) with median age 46 years (IQR 32–57) who use yoga one time per week (62.3%). Yoga is associated with lower systolic (− 2.8 mmHg, standard error 0.6; *p* < .001) and diastolic (− 1.5 mmHg, standard error 0.5; *p* = 0.001) blood pressures. Patients using yoga have 85% increased odds (OR 1.85, 95% CI 1.39–2.46) of having normal blood pressure relative to yoga non-exposed patients. Patients aged 40–59 years have 67% decreased odds (0.33, 95% CI 0.14–0.75) of having stage II hypertension. All effect sizes are age-dependent.

**Conclusions:**

Yoga, as used by patients in their daily lives, may be an effective strategy for blood pressure control and the prevention of hypertension at the population level.

**Supplementary Information:**

The online version contains supplementary material available at 10.1186/s12889-022-12569-3.

## Introduction

Hypertension is the leading modifiable risk factor for cardiovascular disease and related mortality in populations around the world [1]. Adverse cardiovascular events track blood pressure in linear or log-linear patterns [2, 3]. In 2017, after extensive review of the scientific literature on human subjects, the American College of Cardiology and American Heart Association (ACC/AHA) task force recommended lowering the target for healthy, normal blood pressure to below 120 mmHg systolic blood pressure (SBP) and below 80 mmHg diastolic blood pressure (DBP) to mitigate the risk of adverse cardiovascular events in the population [4]. Under these guidelines, prevalence of hypertension is 43.6% in the US population, up from 31.9% under the previous guidelines, and lifetime risk for developing hypertension is estimated to be upwards of 75%; incidence increases with age [5, 6].

An individual’s risk of developing hypertension and/or coronary artery disease has a genetic component, but lifestyle, as an independent risk factor, plays an outsized role. A favorable lifestyle, defined as a healthy diet, regular physical exercise, absence of current cigarette smoking, and absence of obesity, can mitigate the effects of a high risk genetic profile [7, 8]. Likewise, an unfavorable lifestyle can negate the protective effects of a low risk genetic profile. As such, lifestyle modifications are recommended to treat newly diagnosed and early stage hypertension, before initiating a drug regimen [4].

Yoga, a lifestyle-based practice that incorporates physical postures, breath control and/or meditation, has antihypertensive effects in clinical trial settings. Systematic reviews and meta-analyses have demonstrated that yoga reduces blood pressure relative to usual care, in both healthy patients and patients with hypertension or other risk factors for cardiovascular disease [9–11]. In these studies, yoga is as effective as exercise, and can augment the effects of medication [10, 11]. Reported effect sizes depend on the type of yoga, baseline blood pressures, and the methodological quality of the study [9].

In 2017, an estimated 46.5 million adults in the US reported using yoga in the prior 12 months [12]. According to a National Health Statistics Report, individuals primarily use yoga for general wellness and disease prevention, and consistently report knock-on effects that reinforce an antihypertensive lifestyle including, increased exercise, healthier eating, and cutting back or stopping cigarette smoking or alcohol use [13]. But little is known about the actual effectiveness of yoga as used by patients in the real world at controlling blood pressure, because capturing reliable data on lifestyle-based interventions linked to health outcomes in real-world environments is complex, difficult, and seldom prioritized.

Here, we leveraged the electronic health record (EHR) as a source of real-world, observational data on patient lifestyle, blood pressures, and prescribed medications and used mixed effects modeling to assess the antihypertensive effects of yoga as used by patients in their daily lives.

## Methods

### Study design and data sources

We designed an observational, retrospective case control study using EHRs from the University of Pennsylvania Health System, a large, regional health care system comprised of six hospitals and hundreds of outpatient centers in southeast Pennsylvania. The data were sourced from a data warehouse containing repositories of structured and unstructured EHR data maintained by and accessed through the Data Analytics Center at the Perelman School of Medicine at the University of Pennsylvania.

During the study period there were approximately 1.2 million patients in the database, 2–3% of which had at least one clinical chart note containing the word “yoga” [14]. Available structured data fields include demographics, clinical encounters, diagnostic and procedure codes, laboratory test results, and prescribed medications. Unstructured data includes clinical chart notes and imaging data.

### Yoga annotation

Yoga notes were manually annotated and associated patient encounters were only included if the patient had a regular yoga practice, defined here as at least one yoga session per week, at the time of the clinical encounter (e.g., “goes to yoga class on Saturdays”, “exercise: yoga 3x per week”, “does yoga daily at home”). Yoga patient encounters were excluded if the note indicated yoga was used less frequently than one time per week (e.g., “yoga 2-3x a month”) or if there was no indication of frequency (e.g., “exercise: walking, yoga”). Otherwise, notes were annotated by the number of times a patient reported using yoga each week.

### Study population

Because we do not have direct access to the EHR data, we requested a preliminary data set from the Data Analytics Center that was generated by doing a keyword search for “yoga” in the patient chart notes to identify a potential yoga exposed group (see Yoga Annotation subsection), and a yoga non-exposed group; the latter had no mention of “yoga” in their charts and were matched at random to the yoga exposed group on age, sex, and race. A 3:1 non-exposed to exposed ratio was used to capture unmeasured and unknown confounders [15]. This preliminary data set is the basis for this study.

Covariates were chosen to represent biological, behavioral, environmental, and social factors that may affect blood pressure or use of yoga including: age, sex, race, ethnicity, BMI, insurance status, zip code at home address, alcohol use disorder, smoking status, comorbidities common to hypertension, and prescriptions for blood pressure lowering drugs.

Comorbidities are the chronic condition dyads that included hypertension from the Centers for Medicaid and Medicare Services. The list includes: diabetes, hyperlipidemia, chronic kidney disease, heart failure, heart failure and chronic kidney disease, and coronary artery disease A list of ICD codes can be found in Additional File 1. Blood pressure lowering drugs include: antihypertensives, beta blockers, calcium channel blockers, and diuretics. Renin-angiotensin inhibitors were categorized as antihypertensives. A list of prescribed drugs by class can be found in Additional File 2.

Missing data were imputed only within a patient’s own records. Height was imputed as the median across all encounters. Weight was imputed as weight recorded at the nearest encounter within 365 days. (Height and weight were used to calculate the BMI as the weight in kilograms divided by the height in meters squared.) Race and ethnicity were imputed as multiracial or Hispanic if more than one race or ethnicity was reported across the entire record. Insurance status and zip code were imputed as the values recorded at the nearest encounter. Comorbidities, smoking status, alcohol misuse disorder, and blood pressure lowering drugs were assigned to an encounter when documented within the prior 12 months.

Inclusion criteria at the patient level required 3 years of clinical history. There were no set exclusion criteria at the patient level, but all encounters for one patient were excluded because they had an equal number of visits as male and female.

Inclusion criteria at the encounter level required encounters to be with primary care providers and that the providers had seen patients in both the yoga exposed and yoga non-exposed groups, the recorded age of the patient had to be between 18 and 79 years for the encounter to be retained, and blood pressure had to have been recorded at the encounter.

Exclusion criteria at the encounter level were applied after imputation and included: a diagnostic code for pregnancy or end-stage renal disease, weight below the first percentile or above the 99th percentile (< 101 lb. or > 322 lb), missing BMI, or systolic blood pressures greater than 220 mmHg or less than 60 mmHg and diastolic blood pressures greater than 140 mmHg or less than 40 mmHg (ranges indicative of a hypertensive or hypotensive crisis).

All data used in this study were collected during routine care at outpatient visits between November 15, 2006 – November 16, 2016.

### Data set balancing

We used the coarsened exact matching (CEM) package in R to balance the data set by the measured covariates (listed above) [16]. CEM stratifies the data set based on categorical or coarsened values of the covariates, then removes any strata that do not contain at least one case and one control. This ensures there is at least one near match for each observation. Age was coarsened to three predefined age groups: 18–39 years, 40–59 years, and 60–79 years, consistent with the most recent National Center for Health Statistics hypertension report [17]. BMI (kg/m^2^) was coarsened to five categories: underweight (< 18), normal weight (18 < = BMI < 24.9), overweight (24.9 < = BMI < 29.9), obese (29.9 < = BMI < 34.9), and severely obese (> = 34.9), and the remaining covariates were already categorical. CEM generates a weight for each observation as a normalized ratio of cases to controls within each strata, these weights were used in all statistical models. In the context of this study, the yoga exposed are “cases”, and yoga non-exposed are “controls”.

### Statistical analysis

To calculate the effect of yoga on blood pressure, we fit linear mixed effects models with systolic or diastolic blood pressure as the dependent variable. The statistical units in this study are the encounters. Patients may be represented in the data set more than once; each encounter with the health care system that met the inclusion criteria was retained. An encounter is considered a yoga exposure if the clinical chart notes indicate yoga was being used at least one time per week at the time of the encounter (see Yoga Annotation). We included in the model as covariates: age, sex, race, ethnicity, BMI, frequency of yoga practice, insurance status, zip code at home address, diagnostic codes retained after matching (i.e., smoking status, coronary artery disease, diabetes, and hyperlipidemia), and prescriptions for antihypertensives, beta blockers, calcium channel blockers, and diuretics. A random effect term was included for the patient identifiers to create a shared random intercept across all encounters for each individual patient. Each observation was weighted based on the CEM output. To test for age dependent effects, we repeated the analysis within three age groups, 18–39 years, 40–59 years, and 60–79 years. Beta coefficients are presented as effect sizes and statistical significance was set at *p* < 0.05.

To quantify the association between regular use of yoga and four blood pressure categories, defined in the 2017 ACA/AHA blood pressure guidelines, we fit mixed effects logistic regression models with blood pressure category as the dependent variable. Blood pressure categories are: normal (systolic less than 120 *and* diastolic less than 80 mmHg), elevated (systolic between 120 and 129 *and* diastolic less than 80 mmHg), stage I hypertension (systolic between 130 and 139 *or* diastolic between 80 and 89), and stage II hypertension (systolic at least 140 *or* diastolic at least 90 mmHg) [4]. Covariates, the random effect term, and age-based subset analysis are described above. Beta coefficients were exponentiated to generate odds ratios and statistical significance was set at *p* < 0.05.

All data processing was done with Python version 3.6.5.

All statistical analyses were done in R version 3.5.3. For linear mixed effects models we used the lmer function in the lmerTest package and for mixed effects logistic regression models we used the glmer function in the lme4 package [18, 19].

This study was approved by the IRB at the University of Pennsylvania under protocol #826329. Informed consent requirements were waived because the study design is retrospective and observational; patients were not contacted.

Data analysis was conducted between September 2020 – April 2021.

## Results

**Fig. 1:**
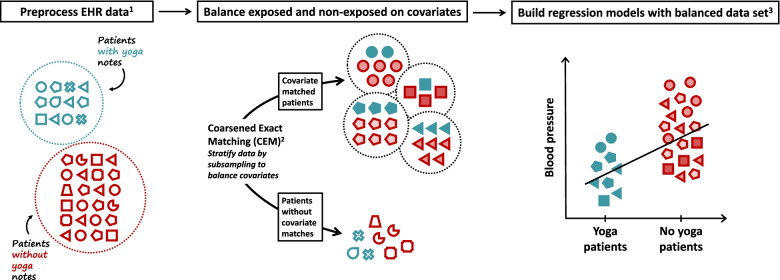
Diagram of the workflow: using electronic health record data to assess the effects of yoga on blood pressure in the real world. The analysis plan is illustrated in three parts, preprocessing, stratification, and modeling. ^**1**^Preprocessing steps included: within patient imputation of covariates (covariates selected to represent biological, behavioral, environmental, and social factors that may affect blood pressure or use of yoga) and the application of inclusion criteria (3 years of medical history, encounters with primary care providers, age 18–79 years) and exclusion criteria (missing BMI, pregnancy or end-stage renal disease, blood pressure and weight thresholds), see Methods. ^**2**^CEM stratifies the data set based on categorical or coarsened values of the covariates, then removes any strata that do not contain at least one case and one control. This ensures there is at least one near match for each observation and each observation is weighted based on the number of cases and controls within its stratum. The symbols in the diagram are colored to indicate different weights. ^**3**^Mixed effects linear and logistic regression models were fit to the balanced data set. Models included all covariates and CEM derived weights

Using a quasi-experimental study design and EHR data, we investigated the effects of yoga on blood pressure in the real world. A diagram of the workflow is presented in Fig. 1.

### Data set characteristics

The preliminary data set, after imputation (Supplementary Table 1, Additional File 3) and applying exclusion criteria (See Supplementary Fig. 1, Additional File 3), had 2755 encounters among 1953 yoga exposed patients and 248,370 encounters among 31,690 yoga non-exposed patients. To this data set, we applied coarsened exact matching (CEM) at the encounter level to generate a covariate balanced data set. CEM matched two-thirds (65.9%) of eligible yoga exposed patient encounters generating a balanced data set of 1815 encounters among 1355 yoga exosed patients and 40,326 encounters among 8682 yoga non-exposed patients. Characteristics of the matched and unmatched yoga exposed patient encounters are shown in Supplementary Table 2, Additional File 3. Characteristics of the balanced data set, at the encounter level, are presented in Table 1.

**Table 1:** Characteristics of covariate matched patients with and without a yoga practice as recorded in the EHR

Encounter level characteristics^**a**^	Patient encounters, No. (%)	
	Yoga (***n*** = 1815)	No yoga (***n*** = 40,326)
**Age, y**		
18–39	700 (38.6)	19,119 (47.4)
40–59	743 (40.9)	17,176 (42.6)
60–79	372 (20.5)	4031 (10.0)
Median (IQR), y	46 (32–57)	41 (30–52)
**Sex**		
Female	1593 (87.8)	37,297 (92.5)
Male	222 (12.2)	3029 (7.5)
**Race**		
Asian	41 (2.3)	564 (1.4)
Black or African American	95 (5.2)	2043 (5.1)
Other^**b**^	82 (4.5)	710 (1.8)
White	1597 (88.0)	37,009 (91.8)
**Ethnicity**		
Hispanic	14 (0.8)	27 (0.1)
**Yoga sessions per week**		
0	NA	40,326 (100)
1	1130 (62.3)	NA
2	351 (19.3)	NA
3+	334 (18.4)	NA
Median (IQR), sessions	1 (1–2)	NA
**Body Mass Index (BMI, kg/m**^**2**^**)**		
Underweight (< 18)	19 (1.0)	46 (0.1)
Normal weight (18–24.9)	1049 (57.8)	25,312 (62.8)
Overweight (24.9–29.9)	532 (29.3)	11,415 (28.3)
Obese (29.9–34.9)	154 (8.5)	2392 (5.9)
Severely obese (> 34.9)	61 (3.4)	1161 (2.9)
Median (IQR), kg/m^2^	24.1 (21.7–26.9)	23.7 (21.6–26.7)
**Behavioral factors**		
Smoking status, smoker	15 (0.8)	71 (0.2)
**Comorbidities**^**c**^		
Coronary Artery Disease	1 (0.1)	4 (0.0)
Diabetes	13 (0.7)	28 (0.1)
Hyperlipidemia	396 (21.8)	4706 (11.7)
**Prescribed drugs**		
Antihypertensives	75 (4.1)	369 (0.9)
Beta blockers	28 (1.5)	116 (0.3)
Calcium channel blockers	6 (0.3)	10 (0.0)
Diuretics	36 (2.0)	191 (0.5)
**Insurance status**		
Medicaid	10 (0.6)	236 (0.6)
Medicare	192 (10.6)	2069 (5.1)
Commercial	1602 (88.3)	37,898 (94.0)
Not recorded	11 (0.6)	123 (0.3)
**Blood pressure category**^**d**^		
Normal	947 (52.2)	20,797 (51.6)
Elevated	266 (14.7)	5275 (13.1)
Stage I hypertension	479 (26.4)	11,349 (28.1)
Stage II hypertension	123 (6.8)	2905 (7.2)
**Blood pressure**		
Systolic, median (IQR), mmHg	116 (108–122)	115 (108–122)
Diastolic, median (IQR), mmHg	72 (68–80)	72 (68–80)
**Records per patient**		
Range, counts	1–16	1–52
Median (IQR), counts	1 (1–1)	3 (1–6)

^**a**^ Individuals may be represented by more than one encounter. To control for non-independent

observations, patient identifiers are modeled as a random effect in all statistical analyses

^**b**^ Other includes: American Indian or Alaskan Native, Native Hawaiian or other Pacific Islander, multiracial, and unknown

^**c**^ No patients with chronic kidney disease or heart failure were retained after matching

^**d**^ Blood pressure categories determined by 2017 ACC/AHA guidelines [4]

Yoga patients are predominantly non-Hispanic (99.2%), white (88.0%), females (87.8%), with a median age of 46 years (IQR 32–57 years), who reported using yoga one time per week (62.3%) (Table 1).

The yoga exposed and yoga non-exposed groups both had normal median BMI (24.1 kg/m^2^ and 23.7 kg/m2) and blood pressures (SBP, DBP: 116 (IQR 108–122), 72 (IQR 68–80) and 115 (IQR 108–122), 72 (IQR 68–80) mmHg. After matching, few patients had common comorbidities of hypertension or prescriptions for blood pressure lowering drugs. Most clinical encounters were linked to commercial insurance, 88.3 and 94.0%, for yoga exposed and yoga non-exposed encounters, respectively.

### Yoga and blood pressure

To calculate the effect of yoga on blood pressure, we used mixed linear regression modeling. In this data set, use of yoga one or more times per week was associated with lower SBP (− 2.8 mmHg, standard error 0.6, *p* < .001) and lower DBP (− 1.5 mmHg, standard error 0.5; *p* = 0.001) than no use of yoga (Table 2). Because blood pressure generally increases with age, we also did a subset analysis to evaluate the effect of yoga on blood pressure within three predefined age groups [17]. Results show age-dependent effects. The average treatment effect of yoga on SBP was smallest and not statistically significant in patients aged 18–39 years (− 1.2 mmHg, standard error 0.9; *p* = 0.152), and increased with age from patients aged 40–59 years (− 3.2 mmHg, standard error 1.0; *p* = 0.002) to patients aged 60–79 years (− 6.4 mmHg, standard error 2.0; p = 0.001). The average treatment effect of yoga on DBP was smallest and not statistically significant in patients aged 18–39 years (− 0.94 mmHg, standard error 0.7; *p* = 0.159), and increased with age from patients aged 40–59 years (− 1.9 mmHg, standard error 0.7; *p* = .008) to patients aged 60–79 years (− 2.4 mmHg, standard error 1.2; *p* = 0.05), the latter only bordering on statistical significance.

**Table 2:**
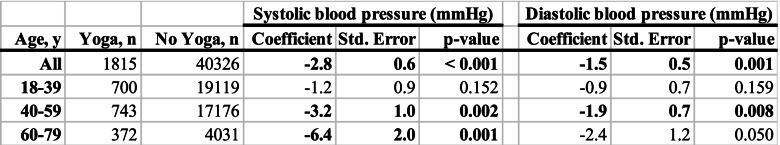
Linear mixed effects modeling shows yoga is associated with lower systolic and diastolic blood pressures

Models adjusted for age, sex, race, ethnicity, BMI, frequency of yoga practice, diagnostic codes retained after matching (i.e., smoking status, coronary artery disease, diabetes, and hyperlipidemia), prescriptions for antihypertensives, beta blockers, calcium channel blockers, and diuretics, insurance status, and zip code at home address with a random effect term for patient identifiers

Effect sizes are robust to variable yoga non-exposed-to-yoga exposed encounter ratios and the composition of the yoga non-exposed encounters (Supplementary Fig. 2, Additional File 3).

### Yoga and blood pressure category

**Fig. 2:**
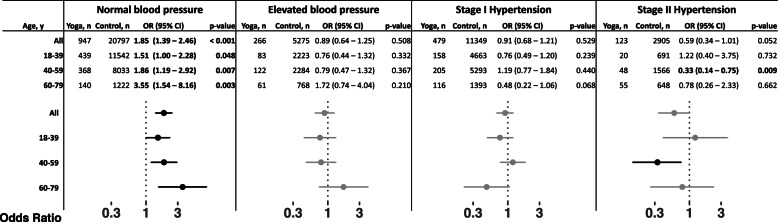
Mixed effect logistic regression modeling quantifies the association between yoga and blood pressure category. Models adjusted for age, sex, race, ethnicity, BMI, frequency of yoga practice, diagnostic codes retained after matching (i.e., smoking status, coronary artery disease, diabetes, and hyperlipidemia), prescriptions for antihypertensives, beta blockers, calcium channel blockers, and diuretics, insurance status, and zip code at home address with a random effect term for patient identifiers

Given that blood pressure is assessed categorically, we also evaluated the relationship between yoga and blood pressure category. Category thresholds for normal blood pressure, elevated blood pressure, stage I hypertension, and stage II hypertension were taken from the 2017 ACC/AHA report [4]. We used mixed effects logistic regression modeling to calculate odds ratios for each blood pressure category given regular use of yoga (Fig. 2). The odds of having normal blood pressure and using yoga were 85% greater than the odds of having normal blood pressure and not using yoga (OR 1.85, 95% CI 1.39–2.46). Subset analysis by age group showed the odds ratios increase with age. Accordingly, the odds of having normal blood pressure and using yoga were 51% greater (OR 1.51, 95% CI 1.00–2.28) in patients aged 18–39 years, 86% greater (OR 1.86, 95% CI 1.19–2.92) in patients aged 40–59 years, and 255% greater (OR 3.55, 95% CI 1.54–8.16) in patients aged 60–79 years than the odds of having normal blood pressure and not using yoga in each age group, respectively.

We observed no association between regular use of yoga and elevated blood pressure or stage I hypertension (Fig. 2). We observed no association between regular use of yoga and stage II hypertension across all patients or in patients aged 18–39 or 60–79 years. For patients aged 40–59 years, the odds of having stage II hypertension and using yoga were 67% less (0.33, 95% CI 0.14–0.75) than the odds of having stage II hypertension and not using yoga.

## Discussion

Hypertension is the leading surrogate marker for cardiovascular diseases and prevalence of hypertension progressively increases with age in the general population [20]. Here, we show use of yoga in daily life is associated with lower blood pressure and with increased odds of maintaining normal blood pressure with advancing age. The implication is that yoga may reduce incidence of adverse cardiovascular events at the population level.

The effect sizes we report in this observational study are consistent with those reported in a meta-analysis across 41 randomized controlled trials and 15 non-randomized controlled trials that estimated yoga reduces SBP by 2.6 mmHg and DBP by 1.6 mmHg in individuals with normal blood pressure at baseline [9]. Reductions in blood pressure of this magnitude are linked to substantial decreases in associated adverse cardiovascular events. A hypothetical incidence study estimated a 2 mmHg reduction in SBP at the population level, in middle-aged patients, would prevent 12,419 incident coronary heart disease events, 7522 incident stroke events, and 18,676 incident heart failure events, annually [21]. Another study estimated a 2 mmHg reduction in DBP at the population level would reduce incidence of coronary heart disease by 6–9% and incidence of stroke by 15% [22]. Modest reductions in blood pressure at the population level will have a greater impact on reducing cardiovascular disease than large reductions in blood pressure of individual patients targeted for intervention [23].

Leveraging the EHR as a source of real-world behavioral data to conduct a large-scale observational study is a key strength of this work. Observational studies fill an important knowledge gap between the effects of an intervention under ideal circumstances, in well-controlled trials, and the effects of an intervention in real world, uncontrolled environments [24]. In the real world, the way individuals integrate health behaviors into their daily life is unlikely to fit the constraints of a clinical trial, for example, in our study, most yoga patients used yoga one time per week, less frequently than the average 4.2 sessions per week used in clinical trials evaluating the antihypertensive effects of yoga [9–11]. To study yoga, observational data are essential because, as we have written in the past, yoga and other mind-body practices are a form of personalized medicine [25]. Yoga is not a single entity, it is a broad term used to encompass many styles and evolutions of an ancient practice, and the effects of any yoga variation on any individual patient will be subjective. By using retrospective, observational data, we presume the patients reporting a regular yoga practice to their health care providers have adopted a style of yoga, a teacher, and a practice environment that resonates with them. In this way, our analysis complements the clinical trial literature by demonstrating that the effects of yoga on blood pressure in clinical trial settings translate to a general patient population, using yoga of their own volition, in the real world.

Demographically, patients with a documented yoga practice in the EHR are similar to those who reported using yoga in a recent National Center for Health Statistics survey, namely they are young and middle-aged, non-Hispanic, white, women [12]. Barriers to broadening this population include, cultural influences, community sentiment, and unfavorable preconceptions of yoga [26, 27]. General barriers to adopting and maintaining a yoga practice, include, time commitment, location, and cost [28–30]. But for patients who experience adverse side-effects to drugs, those concerned with drug-drug interactions or who prefer non-pharmacological treatments, and the growing number of patients who already use yoga, this study provides evidence to support a regular yoga practice as an effective intervention to manage blood pressure.

### Limitations

First, EHR data are not collected for research, and secondary use of the data may introduce unforeseen bias into analyses [31–33]. Second, most mentions of yoga are ambiguous, few notes include quantitative indicators like sessions per week or minutes per session, even fewer describe the type of yoga practice which may be important as meditation, independent of other aspects of a yoga practice, has a potential role in cardiovascular risk reduction [34]. An additional limitation of the yoga mentions is the possibility of social desirability bias, individuals may exaggerate their health behaviors to satisfy a conscious or subconscious need to be viewed favorably by their health care providers. Third, blood pressures may be inaccurate or imprecise due to variable measurement protocols, instrument calibration, or phenomena like white coat hypertension. Fourth, we documented medications as prescribed, but we could not confirm patients were taking medication at the time of a blood pressure reading. Similarly, we used ICD codes to document diagnoses recognizing these are billing codes and not necessarily an accurate representation of a patient’s condition. Lastly, we have modeled average treatment effects in this study, but we fully expect future analyses will reveal yoga to have patient-dependent effects on blood pressure.

## Conclusion

Blood pressure control at the population level is the most effective way to prevent adverse cardiovascular events. This observational, case-control study demonstrates that yoga is associated with lower systolic and diastolic blood pressures, with increased odds of having normal blood pressure, and with decreased odds of having stage II hypertension in patients aged 40–59 years in a general patient population. Yoga, as used by patients in their daily lives, can be an effective strategy for blood pressure control and the prevention of hypertension at the population level.

## Supplementary Information

**Additional file 1.** List of ICD codes. ICD codes of comorbidities most common to hypertension and identified in this data set.

**Additional file 2.** List of drugs. Antihypertensives, beta blockers, calcium-channel blockers and diuretics identified in this data set.

**Additional file 3.** Supplementary methods, results, and discussion.

## Data Availability

The dataset generated and analyzed during the current study is not publicly available because it contains private health information that cannot be de-identified. Questions about the data should be directed to jason.moore@csmc.edu.

## Abbreviations

SBP: Systolic blood pressure
DBP: Diastolic blood pressure
BMI: Body mass index
EHR: Electronic health record
CEM: Coarsened exact matching
