# Supplementary material for: Antihypertensive effects of yoga in a general patient population: real-world evidence from electronic health records, a retrospective case-control study
Source: BMC Public Health. 2022 Jan 27;22:186. doi: 10.1186/s12889-022-12569-3 (PMC8796468; doi:10.1186/s12889-022-12569-3)
Supplement: Supplementary file 3 — Additional file 3. Supplementary methods, results, and discussion. [file 12889_2022_12569_MOESM3_ESM.docx]

Supplementary methods, results, and discussion

**Data set characteristics:**

Yoga patients appear to have fewer encounters with the health care system (Table 1), but this is an artifact of the exclusion criteria for yoga patients; individual encounters that did not mention yoga or frequency of yoga practice were excluded to ensure the patient had a regular yoga practice at the time of the recorded blood pressure (all available encounters were used for imputation and to assign diagnostic codes and prescribed drugs, see Methods).

The distribution of patient characteristics is not equal between the yoga encounters and the control encounters across the entire data set because matching is not one-to-one. CEM stratifies the clinical encounters by the covariates and naturally, the number of yoga patient encounters and control patient encounters within each stratum varies. CEM returns a weight for each encounter based on the normalized ratio of the number of cases to controls within its stratum. All statistical modeling includes the CEM derived weights and a random effect for the patient identifiers to account for nonindependence when a patient is represented in the data set by more than one encounter.

Notably, some characteristics are under-represented in the matched data set, including yoga patients who are non-White, Hispanic, severely obese, have common comorbidities of hypertension, prescriptions for blood pressure lowering drugs, or blood pressure in the range of stage II hypertension. Patients with these characteristics are a minority of the total eligible yoga patient population and their exclusion is linked to zip codes. We found that excluding zip code at home address from the matching criteria would have increased the retention rate of yoga encounters to 96.5%, however, we included zip code because it may be a surrogate for other relevant but unmeasured factors like socio-economic status, environmental exposures, or access to yoga (e.g., distance to a yoga studio or access to high speed internet).

**Effect sizes and control-to-case ratios:**

The effect sizes are robust to changes in the control-to-case ratio and the composition of the controls, both of which can influence observed effect sizes in matched case-control studies (Figure S2).^1^ The control-to-case ratio specifically can determine the statistical power to detect a true effect. The full data set has a control-to-case ratio of approximately 22:1 across all patients.

To explore the relationship between control-to-case ratios and effect sizes in this data set, we subsampled the control encounters at random in ratios of 5, 10, 15, or 20 times the number of yoga encounters (i.e., 22.5%, 45.0%, 67.5%, and 90% of eligible controls). For each of these ratios, we repeated the subsampling, matching, and modeling steps 1000x to generate a distribution of effect sizes. (Control-to-case ratios for sampling were based on the total number of yoga encounters, age-based subsets will have different ratios.)

Across patients of all ages, the median effect size for systolic and diastolic blood pressure are relatively stable, regardless of the ratio. In patients aged 18-39 and 40-59 years, the median effect size decreases as the number of controls increases. In patients aged 60-79 years, the median effect size increases as the number of controls increase. Between ratios, within age groups, all median effect size differences less than 1 mmHg.

As the control-to-case ratio increases, the distributions of effect sizes decrease as expected due to an increase in power and less variation in the composition of controls. This mitigates some concern of bias due to unmeasured confounders. In the real world, it is not possible to isolate the practice of yoga from a lifestyle influenced by a regular yoga practice which may include a healthy diet and regular exercise, behaviors that may or may not be as prevalent in the control group. By subsampling the controls at random and repeating the modeling steps, we see the effect sizes do vary based on the control group, but the median effect sizes are relatively stable. The subset with the fewest patients, those aged 60 - 79 years, show the most variation.

**Selection bias:**

In the Discussion we mentioned secondary use of EHR data may introduce unforeseen biases into an analysis. In this study, we were predominantly concerned with selection bias: selection bias in patient reporting and documentation of yoga in the chart notes, selection bias that may compromises the integrity of the yoga-control comparison, and selection bias in the representation of yoga patients.

To mitigate some of the documentation bias, we restricted the analysis to encounters with clinicians who had written at least one yoga note. To address the comparison bias, we used strict inclusion and exclusion criteria, applied coarsened exact matching to balance the data set, and showed that the median effect sizes are relatively stable as the control group changes in size and composition (Figure S2). Bias in representation of yoga patients may be a limitation of the available data. First, as mentioned above, including zip codes at home address in matching limits the retention rate of yoga patients, understanding why is part of ongoing work. Second, while there are an abundance of yoga notes, most mentions of yoga are ambiguous. Only a small proportion of yoga notes include a quantitative measure like sessions per week. Even fewer specify the type of yoga which may be important as the practice can vary greatly from intensive exercise to slow, methodical movements, with or without breath control and meditation, behaviors that alone or together may affect blood pressure. The eventual capture of standard measures of social and behavioral determinants of health in EHRs, contributed by patients and providers, will be a tremendous boon to the design of future retrospective, EHR-based real-world studies.^2,3^

References

1. Hennessy S, Bilker WB, Berlin JA, Strom BL. Factors Influencing the Optimal Control-to-Case Ratio in Matched Case-Control Studies. *Am J Epidemiol*. 1999;149(2):195-197. doi:10.1093/oxfordjournals.aje.a009786

2. Adler NE, Stead WW. Patients in Context — EHR Capture of Social and Behavioral Determinants of Health. *N Engl J Med*. 2015;372(8):698-701. doi:10.1056/NEJMp1413945

3. Mafi JN, Gerard M, Chimowitz H, Anselmo M, Delbanco T, Walker J. Patients Contributing to Their Doctors’ Notes: Insights From Expert Interviews. *Ann Intern Med*. 2018;168(4):302. doi:10.7326/M17-0583

4. Whelton PK, Carey RM, Aronow WS, et al. 2017 ACC/AHA/AAPA/ABC/ACPM/AGS/APhA/ASH/ASPC/NMA/PCNA Guideline for the Prevention, Detection, Evaluation, and Management of High Blood Pressure in Adults. *J Am Coll Cardiol*. 2018;71(19):e127-e248. doi:10.1016/j.jacc.2017.11.006

|  | **Patient Encounters, No. (%)** | |  | |
| --- | --- | --- | --- | --- |
|  | **Yoga (n = 2,899)** | | **No yoga (n = 268,982)** | |
|  | **pre-imputation** | **post-imputation** | **pre-imputation** | **post-imputation** |
| **Covariate** |  | | | |
| Height | 973 (33.6) | 22 (< 0.1) | 136278 (50.9) | 5018 (1.9) |
| Weight | 93 (3.2) | 8 (< 0.1) | 16733 (6.2) | 1719 (< 0.1) |
| Race | 1 (< 0.1) | 0 (0.0) | 19 (< 0.1) | 0 (0.0) |
| Ethnicity | 59 (2.0) | 57 (2.0) | 1001 (< 0.1) | 749 (< 0.1) |
| Insurance | 635 (21.9) | 70 (2.4) | 73453 (27.3) | 9375 (3.5) |
| Zip code | 6 (< 0.1) | 6 (< 0.1) | 488 (< 0.1) | 487 (< 0.1) |

**Table S1.** Missing data counts before and after imputation. Missing data were imputed at a given encounter using only a patient’s own data from other encounters. Height was imputed as the median across all encounters. Weight was imputed as weight recorded at the nearest encounter within 365 days. Race and ethnicity were imputed based on a patient’s other encounters. If more than one race was reported across the entire history, mixed-race was used. Ethnicity is a binary category in the original data, if a patient had ever been identified as Hispanic, that identity was used. If the ethnicity variable for a given patient was missing at all encounters, we assigned a missing label. Insurance status and zip code were imputed as those recorded at the nearest encounter. Note: the unfiltered source data from which we selected yoga patients has similar rates of missingness to the pre-imputation rates in the no yoga controls.

| **Table S2. Eligible yoga patient encounters, matched and unmatched** | | | | |
| --- | --- | --- | --- | --- |
| **Encounter level characteristics^a^** |  |  |  |  |
|  | **Eligible yoga patient encounters, No.** | | |  |
|  | **Total**  **(n = 2755)** | **Matched**  **(n = 1815)** | **Unmatched**  **(n= 940)** | **Coverage (%)** |
| **Age, y** |  |  |  |  |
| 18-39 | 943 | 700 | 243 | 74.2 |
| 40-59 | 1057 | 743 | 314 | 70.3 |
| 60-79 | 755 | 372 | 383 | 49.3 |
| Median (IQR), y | 48.6 (34.0 – 60.7) | 45.5 (32.2 – 57.2) | 54.9 (38.8 – 65.3) |  |
|  |  |  |  |  |
| **Sex** |  |  |  |  |
| Female | 2310 | 1593 | 717 | 69.0 |
| Male | 445 | 222 | 223 | 49.9 |
|  |  |  |  |  |
| **Race** |  |  |  |  |
| Asian | 97 | 41 | 56 | 42.3 |
| Black or African American | 210 | 95 | 115 | 45.2 |
| Other**^b^** | 209 | 82 | 127 | 39.2 |
| White | 2239 | 1597 | 642 | 71.3 |
|  |  |  |  |  |
|  |  |  |  |  |
| **Ethnicity** |  |  |  |  |
| Hispanic | 60 | 14 | 46 | 23.3 |
| Not recorded | 40 | 0 | 40 | 0.0 |
|  |  |  |  |  |
| **Yoga sessions per week** |  |  |  |  |
| 1 | 1660 | 1130 | 530 | 68.1 |
| 2 | 586 | 351 | 235 | 59.9 |
| 3+ | 509 | 334 | 175 | 65.6 |
| Median (IQR), sessions | 1 (1 – 2) | 1 (1 – 2) | 1 (1 – 2) |  |
|  |  |  |  |  |
| **Body Mass Index (BMI, kg/m^2^)** |  |  |  |  |
| Underweight (< 18) | 38 | 19 | 19 | 50.0 |
| Normal weight (18-24.9) | 1408 | 1049 | 359 | 74.5 |
| Overweight (24.9-29.9) | 846 | 532 | 314 | 62.9 |
| Obese (29.9-34.9) | 304 | 154 | 150 | 50.7 |
| Severely obese (> 34.9) | 159 | 61 | 98 | 38.4 |
| Median (IQR), kg/m^2^ | 24.6 (22.0 – 28.1) | 24.1 (21.7 – 26.9) | 26.3 (22.9 – 30.1) |  |
|  |  |  |  |  |
| **Behavioral factors** |  |  |  |  |
| Alcohol misuse disorder | 13 | 0 | 13 | 0.0 |
| Smoking status, smoker | 48 | 15 | 33 | 31.2 |
|  |  |  |  |  |
| **Comorbidities** |  |  |  |  |
| Chronic kidney disease | 20 | 0 | 20 | 0.0 |
| Coronary artery disease | 22 | 1 | 21 | 4.5 |
| Diabetes | 127 | 13 | 114 | 10.2 |
| Heart failure | 6 | 0 | 6 | 0.0 |
| Hyperlipidemia | 814 | 396 | 418 | 48.6 |
|  |  |  |  |  |
| **Prescribed drugs** |  |  |  |  |
| Antihypertensives | 294 | 75 | 219 | 25.5 |
| Beta blockers | 124 | 28 | 96 | 22.6 |
| Calcium channel blockers | 104 | 6 | 98 | 5.8 |
| Diuretics | 188 | 36 | 152 | 19.1 |
|  |  |  |  |  |
| **Insurance status** |  |  |  |  |
| Medicaid | 35 | 10 | 25 | 28.6 |
| Medicare | 429 | 192 | 237 | 44.8 |
| Commercial | 2232 | 1602 | 630 | 71.8 |
| Self-pay | 1 | 0 | 1 | 0.0 |
| Not recorded | 58 | 11 | 47 | 19 |
|  |  |  |  |  |
| **Blood pressure category^c^** |  |  |  |  |
| Normal | 1248 | 947 | 301 | 75.9 |
| Elevated | 416 | 266 | 150 | 63.9 |
| Stage I hypertension | 811 | 479 | 332 | 59.1 |
| Stage II hypertension | 280 | 123 | 157 | 43.9 |
|  |  |  |  |  |
| **Blood pressure** |  |  |  |  |
| Systolic, median (IQR), mmHg | 118 (110 – 126) | 116 (108 – 122) | 122 (110 – 130) |  |
| Diastolic, median (IQR), mmHg | 74 (68 – 80) | 72 (68 – 80) | 76 (70 – 80) |  |
|  |  |  |  |  |
| **Records per patient^d^** |  |  |  |  |
| Range, counts | 1 - 28 | 1 - 16 | 1 - 26 |  |
| Median (IQR), counts | 1 (1 -1) | 1 (1 -1) | 1 (1 -1) |  |
|  | | | | |
| **^a^** Individuals may be represented by more than one encounter. To control for non-independent  observations, patient identifiers are modeled as a random effect in all statistical analyses.  **^b^** Other includes: American Indian or Alaskan Native, Native Hawaiian or other Pacific Islander, multiracial, and unknown  **^c^** Blood pressure categories determined by 2017 ACC/AHA guidelines^4^  **^d^** Patients may have encounters in the both the matched and unmatched groups | | | | |

**Figure S1.** Flow chart of encounter counts eligible for coarsened exact matching (CEM) based on exclusion criteria. Eligible patient encounters that met the inclusion criteria we retained, covariates were imputed within patients, and then exclusion criteria were applied. **^a^**Diagnoses based on ICD code within the prior 12 months; pregnancy and end-stage renal disease may be documented at the same encounter (i.e., diagnoses are not mutally exclusive). **^b^**Blood pressure is considered extreme when systolic blood pressure is greater than 220 mmHg or less than 60 mmHg and diastolic blood pressure greater than 140 mmHg or less than 40 mmHg; extreme weight was set as exceeding the 1^st^ or 99^th^ percentiles corresponding to < 101 lbs and > 322 lbs.

**Figure S2. Effect size variability by control-to-ratio and control composition.** Controls were subsampled at random in ratios of 5, 10, 15, or 20 times the number of yoga encounters (i.e., 22.5%, 45.0%, 67.5%, and 90% of eligible controls). Boxplots show distributions of the observed effect sizes for 1000 iterations of subsampling, matching, and modeling per ratio. Ratios for sampling were based on the total number of yoga encounters, age-based subsets will have different ratios.
